# Medical Society of the County of Erie

**Published:** 1898-02

**Authors:** F. C. Gram


### MEDICAL SOCIETY OF THE COUNTY OF ERIE.

*Annual Meeting, held January 11, 1898.*

Reported by F. C. GRAM, M. D., Secretary.

THE Medical Society of the County of Erie held its seventy-seventh annual meeting, Tuesday, January 11, 1898, in the rooms of the Buffalo Academy of Medicine, 619 Main street.

The President, Dr. Lucien Howe, called the meeting to order at 9.45 A. M. The minutes of the semi-annual as well as those of the special meeting held to take action on the death of Dr. Edward Storck were read, the former in their entirety and the latter by title only, after which they were approved.

The PRESIDENT thereupon re-appointed the business committee, consisting of Drs. E. H. Long, E. C. W. O'Brien and S. A. Dunham.

Dr. WILLIAM WARREN POTTER, chairman of the committee on membership, reported favorably upon the names of the following candidates for membership and recommended their admission to the society : Drs. Renwick R. Ross, Henry M. Reinhardt, Julius Ullman, Lorenzo Burrows, Katharine S. Munhall, Mary M. Huntley, Edward M. Dooley, William Preiss and D. J. Constantine. The report was adopted and the applicants were thereupon duly elected.

Applications for membership were received from Drs. Christ A. Weinbach, James W. Charters, Francis E. Fronczak, Charles R. Borzilleri, Carro Julia Cummings, E. McC. Pomeroy, N. L. Burnham and John J. Finerty. Referred to the committee on membership.

The treasurer, Dr. EDWARD CLARK, submitted his annual report as follows :

| | |
|---|---|
| Cash on hand as per report for 1896 | $ 199.38 |
| Dues received in 1897 | 332.12 |
| Total resources | $ 531.50 |
| Total disbursements during 1897 | 270.23 |
| Cash on hand January 1, 1898 | 261.27 |
| | $ 531.50 |

The report was referred to an auditing committee, consisting of Drs. Thompson, Preiss and Wetmore, which later announced that the account and vouchers were correct.

The SECRETARY stated that in compliance with the request of the society at the semi-annual meeting, the president and himself had taken the necessary steps to re-incorporate the society, and read the following letter from the attorney who had been intrusted with the work :

BUFFALO, N. Y., August 3, 1897.

FRANKLIN C. GRAM, M. D.,

*Secretary of the Medical Society of the County of Erie, Buffalo, N. Y.*

DEAR SIR—I beg to advise you that the certificate of incorporation of your society was filed in the office of the Secretary of State at Albany on July 29th last, and that the society thereupon became duly incorporated under and by virtue of the laws of the State of New York.

The society retains its original name—Medical Society of the County of Erie—and by that name is capable of maintaining and defending any action or proceeding in all courts and places, and may hold for its own use any real or personal property, including a medical library and apparatus. No change occurs in the management of the society, all officers, censors, and the like, holding over until the next annual election, when their successors shall be elected as has been customary. Regarding the seal, I would suggest that you adopt one pursuant to our conversation of a few days ago.

Yours truly, PETER MAUL.

The suggestion regarding the seal was to the effect that it contain the statements, "organized, 1821, re-incorporated, 1897."

The communication was received and filed.

The following correspondence was then read by the secretary :

ANDREW LANGDON, ESQ.,

*President Buffalo Historical Society,*

MY DEAR SIR—About two years ago I had a conference with you in relation to leaving certain books and documents belonging to the Medical Society of the County of Erie in the care of the Buffalo Historical Society, but no action was taken at that time.

This society was organised seventy-seven years ago. Our old minute books are becoming valuable, and one has even been lost. We are now making a collection of autobiographical sketches, and of photographs of members, which, when completed, will number between 300 and 400. At the request of the president, Dr. Lucien Howe, I desire to ask if the Buffalo Historical Society will act as custodian of these documents, and the like, permitting our members access to them and allowing the Medical Society of the County of Erie to reserve the right of removing the collection if it should so decide at a future date, which might become desirable in case this society secured a permanent home.

Respectfully yours,

FRANKLIN C. GRAM, *Secretary.*

The reply received was as follows :

DR. F. C. GRAM,

*Secretary Medical Society of the County of Erie.*

DEAR DOCTOR—The Buffalo Historical Society will receive, and act as custodian of, the minute books, biographical sketches, portraits, and the like, belonging to the Medical Society of the County of Erie, under the conditions mentioned in your favor of the 6th inst., viz.: that the same shall be deposited with the historical society and remain in its custody until such time as the medical society shall direct otherwise. Individual members shall have access to the same whenever the rooms are open to the public, but neither the collection nor a part of it may be removed except by vote of the medical society. I would suggest that you place the collection in an appropriate case.

Very truly yours,

ANDREW LANGDON, *President.*

On motion, the offer of the Buffalo Historical Society was accepted, and the president authorised to appoint a committee to make the necessary arrangements for the transfer. The president named for such committee, Drs. Gram, Wyckoff and Stockton.

An invitation to attend the annual meeting of the Historical Society was accepted.

Dr. Eli H. Long, chairman of the business committee, presented the program for this session as prepared by the committee.

A committee on nominations was appointed by the president as follows: Drs. Ira C. Brown, W. W. Potter, McPherson, Thornbury and Congdon.

The president called vice-president Coakley to the chair while he delivered his annual address, entitled "Reasons for a law making Credé's method for prevention of ophthalmia of infancy obligatory in public institutions."

On motion of Dr. H. U. Williams, a committee consisting of Drs. S. W. Wetmore, Thornbury and B. G. Long, was appointed to act on the recommendations contained in the address, and on motion of Dr. Dwyer a vote of thanks was tendered the president for his admirable address. Later in the session the committee on the president's address submitted the following report, which the society adopted:

We, the undersigned, a committee appointed to act upon the recommendations for legislation concerning prevention of ophthalmia neonatorum, as set forth in the president's address, do hereby recommend that the President of the Medical Society of the County Erie be authorised personally coöperate with our representatives in the legislature, at Albany, and associate with himself two other members of the society, to constitute a committee, and that every reasonable effort be made to secure enactment of the legislation as set forth in the president's address.

Signed, S. W. Wetmore,
Frank J. Thornbury,
Ben. G. Long.

Dr. J. B. Coakley, chairman of the board of censors, submitted the following report:

*To the Medical Society of the County of Erie:*

The board of censors respectfully presents you with its semi-annual report for the six months ending today as follows :

During the past year the City of Buffalo has been infested with an unusual number of medical quacks and charlatans. All reported cases of this nature have been carefully investigated by this board and several of these illegal practitioners, after having been admonished, have left the city without further trouble. Several cases have been laid before the district attorney by this board, with such evidence as could be procured, but in no case, we regret to say, has there been an indictment found by the grand jury. We cannot but feel that this result might have been different had there been more hearty coöperation with us on the part of the district attorney.

In several of the cases of nonregistered practitioners professing to cure diseases by external applications laid before him, the district attorney decided not even to present the evidence to the grand jury, on the sole authority of a decision rendered by the general term of the supreme court in the case of Smith vs. Lane, reported in 24 Hun, page 632, wherein it was laid down that "Chapter 436 of the Laws of 1874, declaring it to be a misdemeanor for any person to *practise medicine or surgery*, who is not authorised so to do, by a license or diploma from some chartered school, and the like, does not apply to one who undertakes to cure diseases by manipulating the patient's body by rubbing, kneading and pressing it." This case was decided in 1881, since when the medical opinion on the subject has greatly advanced and a new statute, Chapter 398 of the Laws of 1895, has gone into effect. This case besides was never carried to the court of appeals. The courts of other states, notably those of Kentucky, have applied a much wider and broader definition to the term "the practice of medicine." In a very recent case in the latter state, the practice of medicine was defined as follows : "Any person who for compensation, professes to apply *any science* which relates to the prevention, cure or *alleviation* of the diseases of the human body, is practising medicine within the meaning of the statute."

We cannot but believe that the highest court of this state would apply as broad a definition as the Kentucky court, were a case properly brought before it. We urge upon this society at this time the propriety of taking all steps in its power toward the settlement of this very interesting and important question of the construction of the statute of our state specially designed for the prevention of the illegal practice of the healing art, and which in the opinion of this board clearly covers the cases hereinafter enumerated.

It cannot be that the state of New York, with its splendid system of medical education and strict examination for license to practise, will prove itself incompetent to protect its citizens from quackery. The board of censors desires the coöperation of every member of the county society in its efforts to drive out from our midst all forms of the irregular practice of medicine.

As an illustration of what may be accomplished by a combination of proper vigilance and official activity, we may cite the results obtained by the New York county medical society. Within two years after the passing of the law of 1895 against quacks, that society caused the arrest of eighty-three persons for the illegal practice of medicine and obtained convictions in fifty-one cases. These persons paid in fines the sum of $3,690.00 to the society making the complaint, a not inconsiderable source of income following, as a reward, for the strict performance of duty. The New York society further has a large number of cases pending.

If we have the coöperation of our district attorney in the coming year, we feel that a like creditable showing may be made for Erie county. However, unless this society continues a relentless war against these evils, the signs point toward this county's being considered a harbor of refuge for quacks and charlatans driven out from other states, where laws are not only strictly framed, but also strictly enforced.

We present herewith a list of cases investigated, with a statement of the action taken in each as follows :

G. H. A. Schaefer, No. 8 Grey St.—This man does a large advertising business, particularly in an electrical treatment of diseases. He is not registered. The attention of the district attorney was called to him last spring, but no action has yet been taken. Mr. Schaefer has been visited personally by a member of this board and warned to discontinue this practice.

Mrs. Judith Matteson, No. 248 North Division St.—She is the well-known clairvoyant and has never been registered. She was consulted and had charge of the case of one Matilda Limbach, who died on January 28, 1897. We have testimony showing that she prescribed certain medicines *to be taken internally by this patient.* This case was referred to the district attorney, with the evidence mentioned, but action was refused to be taken upon the sole authority of the case of Smith vs. Lane, above referred to. Under the state of facts, which we offered to prove, to-wit : that medicines were prescribed for internal use, we cannot see how the Smith case applies in any way whatsoever.

During the early autumn Buffalo was visited by one Herr Fritz, who advertised himself as "the great modern healer." He was visited by this board and admonished that he was violating the laws of the state, and after a few days' stay left the city. He had previously been driven out of Pennsylvania in like manner.

Theodore F. Vogelgesang, 30 Adams street.—This party does a large advertising business, making a specialty of the application of liniment in diphtheria and rheumatism. These facts can be clearly proven, and were laid before the district attorney, though no action has yet been taken. The doctrine of the Smith case, it seems to us, cannot be held to have any more application to the case of this illegal practitioner than to that of Mrs. Matteson.

The case of Dr. Antonio Sciarrino is one of nonregistration in this state, although he claims to have a regular diploma granted in Sicily. We are in receipt of a letter from the regent's office, at Albany, saying that it is necessary for such a practitioner to be registered in this state, thus corroborating our understanding of the law. This communication, together with the statement of several witnesses, who will prove that Dr. Sciarrino is in active practice at No. 252 Terrace, was laid before the district attorney, but all papers have been returned to us, by him, without action.

In closing our report we, regretfully, are obliged to present a case of the violation of professional ethics by a member of this society.

Dr. ——— * * * * a member of the medical society of the county of Erie, has been for some time engaged in wide-spread advertising, by means of circulars distributed from house to house, and in practising the cure of rupture by means of a secret method known as "The sac lactic method." Dr. ——— is thus violating two sections of the code of medical ethics of the American medical association, to-wit: Sections 4 and 5 of article 1, relating to "The duties of physicians to each other and to the profession at large," which forbid a physician (Sec. 4) to resort "to public advertisements, or private cards, or handbills inviting the attention of individuals affected with particular diseases;" and (Sec. 5) "to hold a patent for any surgical instrument or medicine; or to dispense a secret nostrum, whether it be the composition or exclusive property of himself or of others."

The facts in this case can be abundantly proved, and Dr. ——— told a member of this board, personally, that he cared not what action the medical society of the county of Erie might take with reference to him.

Very respectfully,

J. B. Coakley, *Chairman*,
Irving W. Potter,
Thomas F. Dwyer,
Charles E. Congdon.

Dr. Wyckoff moved to receive the report.

In seconding the motion Dr. W. W. Potter said that the report was thorough and clear, making definite statements that ought to be acted upon. The censors ought to be supported by this society in their efforts to rid the county of itinerent, advertising and all illegal practitioners of medicine.

Dr. Hopkins thought it proper to receive this report as this society had a history in this matter. It touches upon a subject of the most vital interest to its members, viz.: what constitutes the practice of medicine?

Dr. COAKLEY stated that a report received by him from the New York society showed that convictions can be obtained under the law as it stands. That society employs an eminent attorney, and as the fines go to the society the costs are more than covered. Some cases have been carried as far as the court of appeals. The report shows that there have been 124 arrests under the new law, with 74 convictions and 12 cases pending.

On motion of Dr. HOPKINS the censors' report was referred to a committee of three to make recommendations at this session. The chair appointed Drs. Hopkins, Potter and Hoyer.

Later this committee submitted the following, which was adopted:

*To the Medical Society of the County of Erie:*

Your committee to consider the report of the censors begs leave to submit the following:

While the entire report is to be commended, we deem it our more specific duty to recommend the society to grant authority to the censors to employ an attorney, with a view to the prosecution of illegal practitioners of medicine in the county. The district attorney's office appears to be indifferent to this class of work; if this be true and this society expects to obtain convictions it must be at the hands of a special attorney, employed and paid by the society.

The present law seems to be adequate to obtain convictions in the County of New York, and there would seem to be no good reason why it should not be enforced in the County of Erie. Your committee suggests that for the present the expense incurred by the censors for this purpose be limited to $200 for the year 1898.

All of which is respectfully submitted.

HENRY REED HOPKINS, *Chairman.*
FREDERICK F. HOYER,
WILLIAM WARREN POTTER.

Dated Buffalo, January 11, 1898.

The report of the censors was thereupon received and placed on file.

The PRESIDENT called attention to the death of an honorary member of this society, Dr. W. S. Tremaine, and recommended suitable action.

Dr. POTTER moved that a committee be appointed to prepare a memorial. The motion was adopted and the president appointed Drs. Potter, Wyckoff and Thomas Lothrop.

Dr. Roswell Park moved that when the society adjourns it be until 3.30 p. m., in order to give members an opportunity to attend the funeral services, which are to occur at 2.30 p. m. on the same day. Motion adopted.

The committee subsequently reported the following, which was adopted :

### IN MEMORIAM.

### Surgeon William Scott Tremaine, U. S. Army (retired).

Dr. William S. Tremaine died at his home 410 Elmwood avenue, Buffalo, Sunday evening, January 9, 1898, in the sixtieth year of his age. He was born at Charlottetown, Prince Edward Island, Canada, September 13, 1838, went to Boston to live in his early youth, where he received his preliminary education, and took his doctorate degree from the University of Pennsylvania in 1859. He was commissioned assistant surgeon of the 24th Massachusetts Infantry August 7, 1863, and was discharged from that service April 12, 1864. He was appointed surgeon of the 31st U. S. colored Infantry May 2, 1864, which commission he resigned September 9, 1864, to accept a commission as assistant surgeon of U. S. volunteers. He was mustered out of service June 4, 1866, and on June 12th, eight days later, he was commissioned assistant surgeon in the U. S. army with the rank of first lieutenant. He was promoted to captain and assistant surgeon September 16, 1876, and to major and surgeon June 30, 1882. His military service during the civil war was principally with the armies in the field, and after the war ended he was assigned to duty, first at St. John's Hospital at Baltimore, and after several intermediate assignments he became post surgeon at Fort Dodge, Kansas. During service he contracted cholera, yellow fever and several other maladies incident to his military career, and was retired for disability, contracted in the service and in the line of duty, February 27, 1891.

While Dr. Tremaine was stationed at Fort Dodge he became instrumental in organising the Kansas City Medical College, and was appointed professor of surgery in that institution. In 1881, he was assigned to duty as post surgeon at Fort Porter, Buffalo, and while stationed here became engaged in an active private practice, which he has continued to hold until failing health, within the last few months, compelled him to relinquish it. He was one of the active promoters of Niagara University Medical College, and upon its organisation in 1883 he was appointed professor of didactic and clinical surgery in that institution. He was retired to emeritus honors in 1889. During his active career he was surgeon at the Emergency Hospital, which he organised, and he was surgeon-in-chief at the Buffalo Hospital of the sisters of charity. He was also consulting surgeon to the Emergency, St. Francis and the Woman's Hospital, and visiting surgeon to Erie County Hospital. He was a member of the Buffalo Academy of Medicine, having been president of its surgical section, a member of the American Medical Association, comrade of the Grand Army of the Republic, and a companion of the military order of the Loyal Legion of the United States.

Dr. Tremaine was a man of sterling integrity of character, a skilful surgeon and a forceful debater. If he appeared sometimes to be dogmatic it was partly because of his military training, and partly because it was natural for him to be aggressive. He has contributed much to the advancement of the science of medicine and leaves a record as a man and physician to be emulated.

The Medical Society of the County of Erie in respect to the memory of Dr. Tremaine passes the following resolutions:

*Resolved*, That the sympathy of the society is tendered to the widow and children of our deceased colleague in their bereavement.

*Resolved*, That this memorial and these resolutions be spread upon the minutes of this society and that it will attend Dr. Tremaine's funeral in a body.

*Resolved*, That this memorial and resolutions be offered for publication in the BUFFALO MEDICAL JOURNAL and in the daily newspapers.

WILLIAM WARREN POTTER.
CORNELIUS C. WYCKOFF.

The committee on nominations made its report as follows: For president, Dr. Lucien Howe; vice-president, Dr. J. B. Coakley; secretary, Dr. Franklin C. Gram; treasurer, Dr. Edward Clark; librarian, Dr. Wm. C. Callanan. For censors, Drs. J. B. Coakley, C. E. Congdon, T. F. Dwyer, Irving W. Potter and J. F. Krug. For delegates to the Medical Society of the State of New York, Drs. Herbert U. Williams, Arthur W. Hurd, Peter W. Van Peyma, Thomas B. Carpenter, Grover W. Wende, Eugene A. Smith, Earl P. Lothrop, of Buffalo, and J. G. Thompson, of Angola.

Dr. E. H. LONG moved that the report be referred back to the committee to select a vice-president to represent the towns. The amendment was lost.

A motion to adopt the report of the committee and to instruct the secretary to cast the ballot of the society for the nominees was then carried. The secretary cast the ballot of the society for the nominees, whereupon the president declared them duly elected.

On motion of Dr. BROWN the sum of $50 each was ordered to be paid to the secretary and treasurer for their services during the past year.

Treasurer CLARK stated that many members were in arrears with their dues, aggregating to a large amount, among them several members who have advertised their business, and some have refused to pay.

Dr. COAKLEY moved that the treasurer be authorised to employ a collector to assist in securing the dues in arrears.

Dr. BAYLISS moved in amendment that the treasurer make an effort to collect the dues and report the results at the next regular meeting, and that those who refuse to pay be then dropped from the roll of membership. Dr. Coakley accepted the amendment.

Dr. CRONYN raised the point that the by-laws of the society made the necessary provisions.

The resolution as amended was adopted.

Dr. BENEDICT moved that the board of censors in coöperation with the treasurer of this society be requested to report at the next meeting the names of the members of this society engaged in illegal or unethical practice.

The motion was lost.

Dr. CLARK moved that the society direct the treasurer to notify members who are in arrears with their dues for three years or more, that unless they pay the same by the next semi-annual meeting their names may be dropped from the roll, in accordance with the by-laws of this society. Motion adopted.

Dr. KRAUSS stated that the society had appointed a committee to entertain the Central New York Medical Society at its recent Buffalo meeting and had appropriated $50 for this purpose. The number who attended was 87 and far exceeded the number expected. There was, therefore, a deficiency of $37 and he moved that an order be drawn in favor of the Ellicott Club to meet this deficiency.

The motion was carried.

Communication was received from the American Electro-therapeutic Association, which will hold its eighth annual meeting in Buffalo, September 13–15, 1898, asking that a delegate be sent to represent the society and also extending a general invitation to members of this society to attend the sessions. The invitation was accepted and on motion of Drs. KRAUSS and GROSVENOR, President Howe was elected a delegate to the association.

Librarian CALLANAN reported that he received several volumes for the library during the past year and suggested that he be authorised to collect biographical sketches of members who died during the year.

On motion of Dr. CRONYN the report of the librarian was accepted.

The president having been authorised to appoint a committee on legislation to look after the society's interests at Albany, appointed Drs. W. W. Potter, Ernest Wende and Edward Clark as such committee, and re-appointed Drs. E. H. Long, E. C. W. O'Brien and S. A. Dunham as the business committee.

This concluded the business portion of the session and Dr. J. G. THOMPSON, of Angola, read a paper on Colles' fracture.

Dr. GRAM's paper on Infant mortality was read by title and at 12.30 P. M. a recess was taken until 3.30 P. M.

### AFTERNOON SESSION.

Vice-president COAKLEY called the society to order at 3.30 P. M., and Dr. F. H. STANBRO, of Springville, read a paper on Some diarrheal diseases of children.

Dr. WM. G. BISSELL read a comprehensive paper on Formaldehyde disinfection.

He was followed by G. E. GORDON, Esq., of Boston, Mass., who spoke on Modified milk for infant feeding. Although not a physician he said he had a vast experience in the feeding of infants, and the question of how infants should be fed was an important one. It had long since been established that ordinary dilutions of milk did not suffice to feed an infant. It was a choice between artificial food, a wet nurse, and some form of modified milk. He passed over the two former and said the last was the best for the infant which cannot get the mother's breast. After seven years' experience in Boston he found it to be the almost universal opinion among the leading baby feeders, that with good cow's milk, properly prepared according to the physician's prescription, there should be no reason why a child should not thrive. A baby can not only be grown into a fine child, but also children who were handicapped by bad feeding or sickness may be restored by modified milk.

He spoke of the method of producing modified milk, which contains exactly what the physician wants that particular child to get. The success, where properly employed, had been so great that a professor, when recently addressing a post-graduate class, said that when a baby does not do well on laboratory food it was the fault of the physician.

The expense, which is apparently great at fifty cents per day, is little compared to the results and would not pay the laboratory costs were it not for the large amount of milk and cream sold in addition.

Discussion then followed on the papers presented.

Dr. Hopkins said that Dr. Bissell's paper treated of a practical application of an interesting subject, but it seemed to him that the question of formaldehyde disinfection is still in the laboratory stage. The manner of its use is still complicated to a degree, while it should represent simplicity, cheapness and applicability.

Prof. John A. Miller said that experience holds us to a conservative stage. Formaldehyde has an action on microörganisms, but the question of volume strength has been sadly overlooked. He minutely criticised the processes of production by means of the several kinds of apparatus on the market, and asked whether formaldehyde is strictly a germicide or whether it is simply inhibitory in its action.

Dr. Snow, in discussing Mr. Gordon's remarks, said he formerly used a mixture of cream and whey, but considered the Walker-Gordon method an ideal one, because you can prepare the food as needed in every case. Still he did not believe it was a panacea for all children's ills. He differed from Mr. Gordon in the latter's statement that the day of the wet nurse has passed away, for if a proper wet nurse can be obtained the results are the best.

Dr. Eugene A. Smith likewise gave the wet nurse the preference, but said she is usually hard to obtain when wanted. If modified milk will supply the deficiency he would commend it. He related a case in which he had good results by its use.

Dr. Edward Clark said the question of milk is probably attracting more attention of scientific bodies today than any other. He discussed the various steps which the milk undergoes before it reaches the consumer and pointed out where the greatest danger lies. He also touched upon the plan of the Buffalo Health Department, which is designed to modify or remedy the existing evil.

Dr. Sherman briefly discussed the modified milk question, which he considered the best method yet introduced. It fails in some cases, so does mother's milk.

A vote of thanks was tendered Mr. Gordon for his interesting address and to all those who presented papers.

The society at 6 o'clock P. M. adjourned.

